# Short and Long-Term Effects of the Exposure of Breast Cancer Cell Lines to Different Ratios of Free or Co-Encapsulated Liposomal Paclitaxel and Doxorubicin

**DOI:** 10.3390/pharmaceutics11040178

**Published:** 2019-04-11

**Authors:** Marina Santiago Franco, Marjorie Coimbra Roque, Mônica Cristina Oliveira

**Affiliations:** Department of Pharmaceutical Products, Faculty of Pharmacy, Universidade Federal de Minas Gerais, Av. Antônio Carlos, 6627, Belo Horizonte 31270-901, MG, Brazil; marjoriecoimbra@gmail.com

**Keywords:** ratiometric delivery, synergism, nuclear morphometric analysis, senescence detection, clonogenic assay, migration

## Abstract

Background: Associating paclitaxel (PTX) to doxorubicin (DXR) is one of the main chemotherapy strategies for breast cancer (BC) management. Protocols currently available consist in administering both drugs on their maximum tolerated dose, not taking into account the possible differences in efficacy due to their combination ratio. In the present study, the short and long-term cytotoxic effects as well as migratory effects of PTX, DXR, and its combinations at 10:1; 1:1 and 1:10 PTX:DXR molar ratios either free or co-encapsulated in liposomes were evaluated against three human BC cell lines (MDA-MB-231, MCF-7, and SKBR-3). Method: The MTT assay was used to screen for synergy or antagonism between PTX and DXR and the combination index value was calculated using the CalcuSyn software. Nuclear morphological alterations were evaluated by staining the cells with Hoescht 33342. The investigation of senescence and clonogenicity of BC cell lines exposed to different treatments was also studied. In addition, the ability of these cells to migrate was assessed. Results: Taken together, the results presented herein allow us to suggest that there is no benefit in enhancing the PTX concentration above that of DXR in the combination for any of the three cell lines tested. Conclusion: The developed liposomes co-encapsulating PTX and DXR in different molar ratios retained the biological properties of the mixture of free drugs and are valuable for planning new therapeutic strategies.

## 1. Introduction

Breast cancer (BC) is the most frequent cancer among women in both more and less developed regions. It accounts for the most frequent cause of cancer death in women in less developed regions and in more developed regions it comes after lung cancer [1]. The treatments currently available for BC management are allowing for the mortality rates to decline [2]. Among the available treatments, the use of several anthracycline-based regimens increases the benefits, when compared to those obtained from the use of cyclophosphamide, methotrexate, and fluorouracil combinations, hence it is the most effective chemotherapy. It is also known that, the incorporation of taxanes further improves patient outcomes in the neo/adjuvant setting [3,4]. However, the clinical benefits provided by using combined taxanes and anthracyclines for metastatic BC remain uncertain, and the treatment with a single agent is recommended as it has been shown to produce equivalent clinical outcomes and has less toxicity compared to other regimens [5].

Despite the efficacy, the chemotherapy schemes now available lead not only to severe side effects during the treatment, but also after its completion. Of particular importance are the potential long-term sequelae of the cardiotoxicity of anthracycline-based therapy for survivors of breast cancer, which might appear more than 10 years after administration of the regimens. This highlights the need of searching for new therapeutic strategies [6,7]. Nanomedicine brings promising new therapeutic options. Some of them, such as Doxil^®^, a liposomal formulation of doxorubicin (DXR) and Abraxane^®^, albumin bound paclitaxel (PTX) nanoparticles, are already widely a successfully used for clinical treatment of BC [8]. A new approach on nanomedicine that started attracting significant attention in the first decade of this century is the development of nanosystems designed to co-encapsulate drugs in synergistic ratios. That was due to the observation that the same anticancer drug combinations can act synergistically or antagonistically against the same tumor cells in vitro, depending only on the ratios in which these agents are combined. This finding led to the idea that we might be failing to exploit the full potential of the chemotherapy regimens used in the clinics nowadays, since the ratio of the drugs reaching the tumor upon the administration of free drugs is completely arbitrary [9]. Therefore, the need to control drug ratios reaching the tumor site comes as a great challenge, and the ratiometric drug delivery using nanocarriers rises as a promising strategy. This strategy was the focus of two reviews recently published by Franco and Oliveira [10,11].

Liposomal formulations designed according to this strategy have shown to be able to maintain drug ratios in the plasma after injection as well as deliver to the formulated drug ratio directly to the tumor tissue. This ensures that the effect observed in vitro is translated into in vivo, and chemotherapy is optimized [12,13,14,15,16,17,18]. Finally, the idea was validated in the clinics for a liposomal formulation co-encapsulating a fixed ratio of cytarabine:daunorubicin, approved by the Food and Drug Administration (FDA) in 2017, under the trademark of Vyxeos [19,20].

We recently reported the results of antitumor activity and toxicity of long-circulating and fusogenic liposomes co-encapsulating PTX and DXR (LCFL-PTX/DXR) at molar ratio of 1:10, respectively, in mice bearing the 4T1 mammary carcinoma. The LCFL-PTX/DXR presented superior efficacy compared to treatments with free PTX or free DXR, and improved cardiac toxicity profile compared to treatment with the free combination of these drugs, enabling its co-administration [21].

Here we evaluate the short and long-term effects of the exposure of MDA-MB-231, MCF-7, and SKBR-3 human breast cancer cell lines to different ratios of free or liposomal co-encapsulated PTX and DXR. 

## 2. Materials and Methods

### 2.1. Materials

1,2-Dioleoyl-*sn*-glycero-3-phosphoethanolamine (DOPE) and 1,2-distearoyl-*sn*-glycero-3-phosphoethanolamine-*N*-[amino(polyethyleneglycol)-2000 (DSPE-PEG2000) were supplied by Lipoid GmbH (Ludwigshafen, Germany). Cholesterol hemisuccinate (CHEMS), doxorubicin (DXR), 4-(2-hydroxyethyl)-1-piperazineethanesulfonic acid (HEPES) sodium salt, sodium chloride, sodium hydroxide, and Cremophor EL were obtained from Sigma-Aldrich Co. (St. Louis, MO, USA). Paclitaxel (PTX) was supplied by Quiral Quimica do Brasil S.A (Juiz de Fora, Brazil).The human breast adenocarcinoma cell lines MDA-MB-231, MCF-7 and SKBR-3 were purchased from the American Type Culture Collection (Manassas, VA, USA). The different culture media (Dulbecco’s Modified Eagle’s Medium, DMEM; Minimum Essential Medium, MEM and McCoy), fetal bovine serum, penicillin/streptomycin and 3-(4,5-dimethylthiazolyl-2)-2,5-diphenyltetrazolium bromide (MTT) were obtained from Sigma-Aldrich Co. (St. Louis, MO, USA). All other chemicals used in this study were of analytical grade. 

### 2.2. Liposome Preparation

LCFL-PTX/DXR were prepared by the lipid film hydration technique, as described elsewhere [20]. Briefly, DOPE, CHEMS, and DSPE-PEG2000 (in a 5.7:3.8:0.5 molar ratio, respectively) were dissolved in chloroform at a 10 mmol L^−1^ total lipid concentration, mixed with and PTX (0.25 mg/mL) on a round bottom flask and submitted to evaporation under reduced pressure in a 50 °C water bath until a thin lipid film had been obtained. The round bottom flask containing the lipid film was then maintained for 1 h under a chloroform atmosphere for better dispersion of PTX in the lipids, according to the technique known as annealing [22]. A solution of ammonium sulfate (300 mM, pH 7.4) preheated to 50 °C was then added to the film and the mixture was kept in a 50 °C ultrasonic bath for 10 min for its hydration. Non-entrapped PTX was eliminated by centrifugation at 3000 rpm, 25 °C, for 10 min (HeraeusMultifuge X1R centrifuge, Thermo Fischer Scientific, Thermo Fischer Scientific, Waltham, MA, USA). For removing non-entrapped ammonium sulfate, liposomes were maintained on dialysis overnight against HEPES buffered saline (HBS), pH 7.4. PTX concentration in these liposomes was determined by high performance liquid chromatography (HPLC) analysis. After the determination of the PTX concentration, DXR was remotely loaded into liposomes driven by the transmembrane ammonium sulfate gradient in order to obtain a PTX:DXR molar ratios of 10:1; 1:1 or 1:10. For that, the liposomes containing PTX were incubated with DXR for 2 h at 25 °C and then submitted to another dialysis against HBS at pH 7.4, overnight, to remove non-entrapped DXR. Fusogenic liposomes containing only PTX (LCFL-PTX) were prepared as described above without addition of DXR and further steps and fusogenic liposomes containing only DXR (LCFL-DXR) were prepared without the addition of PTX during film formation, and DXR was added to a total concentration of 2 mg/mL. Blank liposomes were prepared without the addition of neither PTX nor DXR.

### 2.3. Liposomes Characterization

#### 2.3.1. Determination of the Diameter, Polydispersity Index, and Zeta Potential

The diameter of the vesicles and the polydispersity index (PI) were determined by dynamic light scattering (DLS). The measurements were performed at a temperature of 25 °C, using a 90° laser incidence angle. The zeta potential (ζ) of the vesicles was determined by DLS associated with electrophoretic mobility. To perform both measurements, the liposomes were diluted in HBS, pH 7 and evaluated on the Zetasizer Nano ZS90 equipment (Malvern, UK).

#### 2.3.2. Determination of the Content of PTX and DXR

PTX and DXR levels were determined by high-performance liquid chromatography (HPLC, Waters Instruments, Milford, MA, USA). For determination of PTX levels, the mobile phase was composed of 55% acetonitrile in water. The elution time was of 8 min, the Hibar 250-4 LiChrospher 100RP-18, 25 cm × 4 mm, 5 μm column (Merck, Darmstadt, Germany) was used. The column was kept at room temperature, the flow rate was set at 1.2 mL/min and the detection wavelength was 227 nm. For determination of the DXR concentration, the mobile phase was composed of methanol:phosphate buffer 0.01 M (65:35, *v*/*v*). The elution time was of 7 min, the ACE^®^ C8 25 cm × 4.6 mm, 5 μm column (Merck, Darmstadt, Germany) was employed. The column was kept at room temperature, the flow rate was set at 1.0 mL/min and the detection was performed with a fluorescence detector model 2475 (Waters Instruments, Milford, MA, USA) with excitation and emission wavelengths of 470 nm and 555 nm, respectively. The PTX and DXR encapsulation percentage (EP) were calculated according to the following equation:

(1) $$EP=\frac{\left[amount\;ofdrug\;in\;purified\;liposomes\right]}{\left[amount\;ofdrug\;in\;the\;non-purified\;liposomes\right]}\times 100$$

### 2.4. Cell Culture

The human breast adenocarcinoma cell lines MDA-MB-231, MCF-7 and SKBR-3 were cultivated in DMEM, MEM supplemented with 0.01 mg/mL insulin and McCoy media, respectively, all supplemented with 10% fetal bovine serum (FBS). All cell lines were cultivated in the presence of penicillin (100 IU/mL) and streptomicin (100 μg/mL) and maintained at 37 °C and 5% CO_2_ in a humidified atmosphere. Prior to the experiments, all cell lines were screened for mycoplasma by polymerase chain reaction (PCR), with negative results. 

### 2.5. In Vitro Screening of PTX and DXR for Synergy and Antagonism

The MTT assay was chosen as the means to screen for synergy or antagonism between PTX and DXR in the different cell lines studied. For that, cells were plated in different densities (6 × 10^3^ cells for MDA-MB-231 cell line; 1 × 10^4^ cells for MCF-7 cell line and 5 × 10^3^ cells for SKBR-3 cell line) per well in 96 well plates, and kept in the incubator for around 24 h prior to exposition to the treatments. To determine the inhibitory concentration of 50% (*IC*_50_) of each free or liposome-encapsulated drug, cells were exposed to a minimum of 8 different concentrations of PTX, DXR, LCFL-PTX, or LCFL-DXR for 48 h. To screen for synergy or antagonism between the drugs, cells were exposed to a minimum of 6 concentrations of different fixed molar ratios (10:1; 1:1; 1:10) of a mixture of free PTX:DXR or LCFL-PTX/DXR (co-encapsulating the different fixed molar ratios) for 48 h. After the incubation time, treatments were removed and 100 mL of MTT (0.5 mg/mL) were added to the wells. The plates were incubated for 2 h at 37 °C. After incubation time, media containing MTT were removed from the wells and 100 mL of DMSO was added to solubilize the formazan crystals. The absorbance of the wells was determined using a bench spectrophotometer model Benchmark Plus (Bio-Rad, Hercules, CA, USA) at 570 nm. All experiments were performed in quadruplicate of wells per concentration and triplicate of plates.

The test data were converted to mean fraction of cell survival relative to untreated cells (control group). The fraction of affected cells (*f*_a_) was subsequently determined for each concentration of treatment and the data sets were analyzed by the median-effect analysis using the CalcuSyn software (version 2.0, Biosoft, Ferguson, MO, USA, year). It uses the combination index (*CI*) value as a quantitative indicator of the degree of synergy or antagonism. Using this analysis method, CI value around 1.0 indicates additive activity, *CI* value >1 indicates antagonism, and a *CI* value <1.0 indicates synergism [23].

Two controls were performed for the MTT assay. The first consisted in verifying the intrinsic biologic activity of the long-circulating and fusogenic liposomes without anticancer drugs (LCFL-blank) and cremophor against the tested cell lines [24,25,26]. Therefore, the different cell lines were exposed to these agents in the same range of concentrations as treatments. The second control consisted in evaluating the possible reduction of the MTT by the studied substances in cell-free wells [27]. In this experiment, cell-free wells received PTX solubilized in cremophor and DXR on a concentration of 100 mM and LCFL-blank in equivalent lipid concentration to that obtained for LCFL-PTX at 100 mM. These concentrations were chosen based on the fact that they were much higher than that used for the cytotoxicity assays. On these experiments, plates were submitted to the same protocol described above. The only difference was that in the experiments with cell-free wells, dimethyl sulfoxide (DMSO) was added directly to the media after incubation with MTT.

### 2.6. Nuclear Morphometric Analyses (NMA)

To evaluate nuclear morphological alterations after treatments, the different cell lines were plated at a density of 2.0 × 10^5^ cells/well in 6-well plates and incubated at 37 °C for 24 h. After incubation time, cells were treated for 48 h with 2 mL of different treatments (PTX, DXR, and the mixtures of free PTX:DXR at 10:1; 1:1 or 1:10 molar ratio) all at a total concentration of 70 nM. Control wells received 2 mL of fresh media. After incubation, cells were fixed with formaldehyde 4% for 10 min. Fixed cells were stained with a Hoescht 33342 (0.2 μg/mL) solution for 10 min at room temperature in the dark. Nuclei fluorescence images were captured using a microscope AxioVert 25 with a fluorescence module Fluo HBO 50 connected to the Axio Cam MRC camera (Zeiss, Oberkochen, Germany). A total of a hundred nuclei per treatment were analyzed using the Software Image J 1.50i (National Institutes of Health, Bethesda, MD, USA, 2016) and the plugin “NII_Plugin” available at http://www.ufrgs.br/labsinal/NMA/.

### 2.7. Senescence-Associated-β-galactosidase (SA-β-gal) Assay

The staining procedure has been performed as described by Debacq-Chainiaux and coworkers [28]. Briefly, the different cell lines (5 × 10^4^ cells) were seeded in 24-well plate and incubated at 37 °C for 24 h. After incubation time, cells were treated for 48 h with 500 μL of different treatments (PTX, DXR, and the mixtures of free PTX:DXR at 10:1; 1:1 or 1:10 molar ratio). All treatments were added at a total concentration of 70 nM. Control wells received 500 μL of fresh media. After treatment, cells were washed with PBS and fixed in 2% formaldehyde (*v*/*v*) and 0.2% glutaraldehyde (*v*/*v*) in PBS buffer for 5 min. Fixative was removed by washing cells with PBS and then cells were stained for 12 hours with X-gal staining solution (0.5 mg/mL X-gal, 40 mM citric acid/sodium phosphate pH 6.0, 5 mM potassium ferrocyanide, 5 mM potassium ferricyanide, 150 mM NaCl, and 2 mM MgCl_2_).

### 2.8. Clonogenic Assay

Clonogenic assay was performed based on one of the protocols described by Franken and coworkers (2006) [29]. MDA-MB-231, MCF-7 and SKBR-3 cells were plated on a density of 2.5 × 10^5^ cells/well in 6-well plates and incubated at 37 °C for 24 h. After incubation time, cells were treated for 48 h with 2 mL of different treatments (PTX, DXR, the mixtures of free PTX:DXR at 10:1; 1:1 or 1:10 molar ratio, LCFL-PTX, LCFL-DXR, or LCFL-PTX/DXR co-encapsulating different fixed molar ratios of 10:1; 1:1 or 1:10) all at a total concentration of 70 nM. Control wells received 2 mL of fresh media. Then, cells were harvested and counted. Different densities of viable cells (300 cells for MDA-MB-231 or MCF-7 cell lines and 900 cells for SKBR-3 cell line) were re-plated in 6 well plates to evaluate the ability of the remaining cells to form colonies. Twenty-one days after re-plating, colonies were fixed with formaldehyde 4% for 10 min and stained with 2 mL of crystal violet for 1 hour. Colonies, consisting of a group of at least 50 cells, were counted using an AxioVert 25 (Zeiss, Oberkochen, Germany) microscope, and the percentage of colony formation relative to the control was calculated for each group.

### 2.9. Migration Assay

To study the bi-dimensional migration of the different cell lines they were plated at a density of 2.0 × 10^5^ cells/well in 12-well plates and incubated at 37 °C for 24 h. Then, a straight wound was made in individual wells with a 10 μL pipette tip. This point was considered the “0 h,” and the “zero wound” was photographed using a microscope AxioVert 25 with a connected Axio Cam MRC camera (Zeiss, Oberkochen, Germany). After obtaining the wounds, control wells received 1 mL of fresh media and the other wells received 1 mL of media containing the different treatments (PTX, DXR, the mixtures of free PTX:DXR at 10:1; 1:1 or 1:10 molar ratio, LCFL-PTX, LCFL-DXR or LCFL-PTX/DXR co-encapsulating different fixed molar ratios of 10:1; 1:1 or 1:10) all at a total concentration of 70 nM. The plates were incubated at 37 °C for 24 h. On these experiments, from the platting moment until the end of the assays cells were kept on starvation, meaning the different media contained only 1% FBS. After incubation cells were fixed with formaldehyde 4% for 10 min. Images along the wounds of control and treated groups were obtained in phase contrast. Wound areas were obtained using the MRI Wound Healing Tool plugin for the free version of Image J 1.45 software (National Institutes of Health, Bethesda, MD, USA). The wound healing percentage was calculating according to the following equation:

(2) $$\%\;wound\;healing=100-\frac{\left(area\;of\;treated\;wound\times 100\right)}{area\;of\;the\;“zero\;wound”}$$

### 2.10. Statistical Analyses

The results were expressed as mean ± SD of three independent experiments. Statistical analyses were performed by one-way analysis of variance (ANOVA) followed by Tukey’s post test. Prior to ANOVA analyses, data were transformed as indicated on the tables to fit the normality and homocedasticity requirements, which were evaluated by Kolmogorov-Smirnov and Levene tests, respectively. Differences were considered statistically significant when *P* values were <0.05. GraphPad Prism 5.04 Software (GraphPad, San Diego, CA, USA) was used to calculate all data.

## 3. Results

### 3.1. Physicochemical Characterization of the Different Liposomal Formulations

Size measurements of the different formulations demonstrated that the encapsulation of PTX, DXR or co-encapsulation of these drugs into LCFP did not affect significantly the size of the vesicles compared to LCFP-blank (*P* > 0.05). The mean diameter of the different formulations ranged from 226.4 to 249.8 nm. Graphical representations of the intensity particle size distribution for the different formulations are provided in Supplementary Materials Figure S1. The mean PDI values varied from 0.27 to 0.33 indicating that the populations of the different formulations have low polidispersity. Zeta potential mean values ranged from −4.40 to −6.86 mV, near to neutrality, as expected for formulations containing PEG on its bilayer [30]. Besides, the use of a gradient to encapsulate DXR allowed for the obtainment of formulations with PTX:DXR molar ratios close to the desired 10:1; 1:1 and 1:10 as shown in Table 1.

### 3.2. In Vitro Screening of PTX and DXR Treatments for Synergy and Antagonism

When evaluated for short-term cytotoxicity, all three cell lines tested were more sensitive to PTX treatment compared to DXR treatment in free form (*P* < 0.05), as summarized in Table 2. The encapsulation of PTX in liposomes did not alter its cytotoxicity against MDA-MB-231 and MCF-7 cell lines (*P* > 0.05), while a reduction on cytotoxicity was observed for SKBR-3 cell line (*P* < 0.05). For all three cell lines, encapsulation of DXR in liposomes did not affect its cytotoxicity (*P* > 0.05). Similarly to the free drugs, LCFL-PTX were more cytotoxic than LCFL-DXR against MCF-7 and SKBR-3 cell lines (*P* < 0.05). No difference in cytotoxicity between LCFL-PTX and LCFL-DXR was observed for liposome-encapsulated drugs against the MDA-MB-231 cell line (*P* > 0.05). 

Concerning cytotoxicity of the treatments with combined PTX:DXR at different molar ratios either free or liposome co-encapsulated, MDA-MB-231 and MCF-7 cell lines presented a similar response profile. For these cell lines, an enhancement of the cytotoxicity was observed with the enhancement of DXR concentration present in the combinations. On the other hand, for the SKBR-3 cell line, treatments with a higher concentration of PTX were more cytotoxic compared to those containing higher concentrations of DXR (Supplementary Materials, Figure S2).

It is well known that the combination effect (synergy, additivity or antagonism) can be affected by the ratio of the drugs in the combination [12]. For this reason, we examined the cytotoxicity through the median-effect analysis using the CalcuSyn software to obtain the *CI* values for the different combinations. Concerning the combination effect for the mixture of free drugs in different molar ratios, synergism (*CI* < 1.0) was observed for the treatment of MDA-MB-231 and MCF-7 cell lines only with PTX:DXR at molar ratio of 1:10, while for the SKBR-3 cell line all molar ratios of combined free drugs led to antagonism (*CI* > 1.0). Different studies have already reported that combination effect profiles might change upon co-encapsulation of the studied drugs [10]. In the present study, upon co-encapsulation in LCFL, except for treatment with PTX:DXR at molar ratio of 1:10 that turned out to be antagonic for the MDA-MB-231 cell line, no changes in the combination effect profiles were observed for the different cell lines.

Control experiments revealed that LCFL-blank and cremophor have no interference in the metabolic activities of the different cell lines as they were assessed on a maximum of 110% and a minimum of 80% compared to that of the control in the evaluated concentration ranges. The absorbances in the cell-free wells exposed to 100 μM of PTX solubilized in cremophor (0.23 ± 0.02), 100 μM of DXR (0.22 ± 0.00) or to LCFL-blank in equivalent lipid concentration to the LCFL-PTX treatment at 100 μM (0.22 ± 0.01) did not vary significantly from that observed for the control group (0.24 ± 0.01; *P* > 0.05) after incubation with MTT. This finding proved that in the present study, the reduction of MTT to formazan occurred only in the presence of cells.

#### Comparison of the Short-Term Cytotoxicities of the Different Combinations of PTX:DXR

For the MDA-MB-231 and MCF-7 cell lines, it was observed that the increase of the DXR concentration in the combinations allowed for an enhancement of the cytotoxicity compared to the PTX:DXR combination at 10:1 molar ratio. Considering combinations of PTX:DXR on the free form, the 1:1 and 1:10 molar ratios were 4.6-fold and 5.4-fold more cytotoxic, respectively, than the 10:1 molar ratio against the MDA-MB-231 cell line. For the MCF-7 cell line, the 1:1 and 1:10 molar ratios were 5.1-fold and 5.7-fold more cytotoxic, respectively, than the 10:1 molar ratio. Considering the co-encapsulated PTX:DXR combinations, the 1:1 and 1:10 molar ratios were 1.3-fold and 2.1-fold more cytotoxic, respectively, than the 10:1 molar ratio against the MDA-MB-231 cell line. For the MCF-7 cell line, the 1:1 and 1:10 molar ratios of the co-encapsulated drugs were 4.1-fold and 6.5-fold more cytotoxic, respectively, than the 10:1 molar ratio. No difference in cytotocicity was observed between 1:1 and 1:10 molar ratios combinations of the free drugs or between 1:1 and 1:10 molar ratios combinations of co-encapsulated drugs against MDA-MB-231 and MCF-7 cell lines (*P* > 0.05).

The SKBR-3 cell line presented a different profile as the PTX:DXR combinations at 1:1 and 1:10 molar ratios of the free drugs were 3.0-fold and 39.0-fold less cytotoxic, respectively, than the 10:1 molar ratio. When PTX:DXR were co-encapsulated in liposomes at 1:1 and 1:10 molar ratios, these combinations were 1.3-fold and 5.9-fold, respectively, less cytotoxic than the PTX:DXR combination at 10:1 molar ratio against the SKBR-3 cell line. The combination of PTX:DXR at 1:1 molar ratio presented higher cytotoxicity compared to the 1:10 molar ratio either in free or co-encapsulated in liposomes against the SKBR-3 cell line (*P* < 0.05).

### 3.3. Nuclear Morphometric Analyses (NMA)

It is known that several cellular processes can be inferred with the analysis of nuclear morphometric features. The Nuclear Morphometric Analysis (NMA) tool, developed by Filippi-Chiela and coworkers (2012), divides nuclei into six groups according to their morphological appearances, each having a putative biological meaning: normal (N), irregular (I, mitotic catastrophe or other nuclear damaging event), small regular (SR, apoptosis in an early or intermediary stage), small (S, mitosis), small irregular (SI, mitosis with damage or nuclear fragments), large regular (LR, senescence) and large irregular (LI, mitotic catastrophe or other nuclear damaging event) [31].

**Figure 1:**
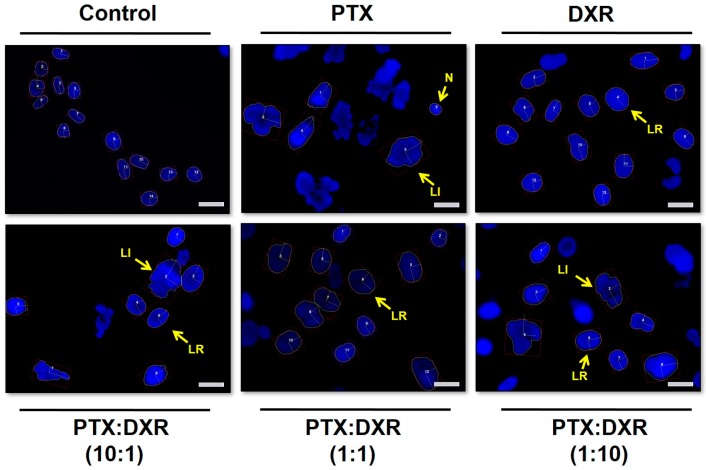
Fluorescence photomicrographs of MDA-MB-231 cell line nuclei stained with Hoescht 33342 after different treatments at concentration of 70 nM for 48 h. Some of the different nuclei morphometric phenotypes observed are indicated. Images are representative of three independent experiments. Amplification 40×, scale bar = 20 μm. Abbreviations: DXR, doxorubicin; PTX, paclitaxel; N, normal; LI, large irregular; LR, large regular.

Figure 1 presents fluorescence photomicrographs of Hoescht 33342 stained MDA-MB-231 nuclei, where nuclear enlargement for cells exposed to the different treatments is evident when compared to untreated cells.

**Figure 2:**
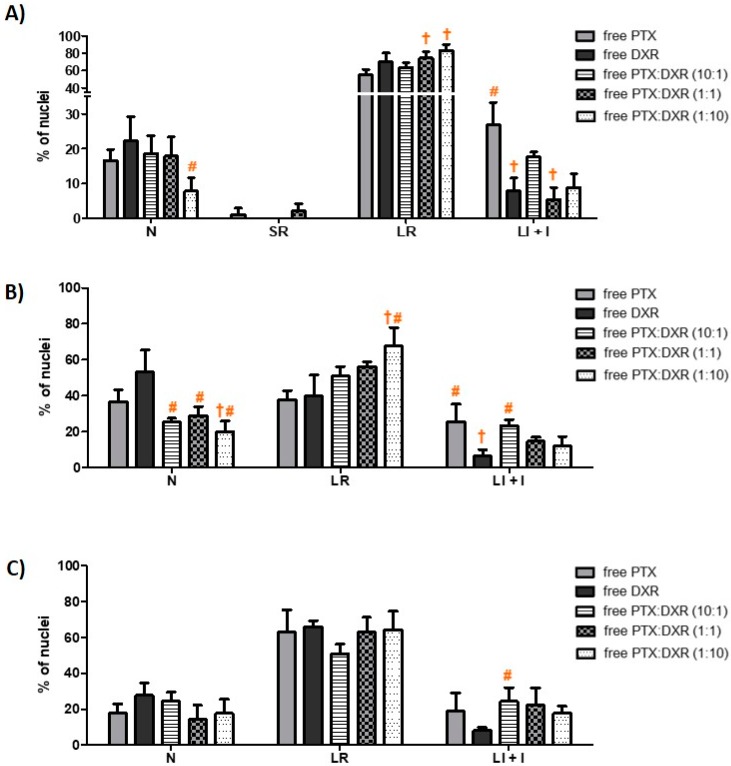
Nuclear morphometric distribution of MDA-MB-231 (**A**), MCF-7 (**B**) and SKBR-3 (**C**) cell line nuclei exposed to 70 nM of different treatments for 48 h. Data shown represent the mean ± SD of three independent experiments. † = differs statistically from the PTX treatment; # = differs statistically from the DXR treatment. Data for MDA-MB-231 and MCF-7 cell lines were transformed as *y* = square root(value) and for SKBR-3 cell line as *y* = log(value) prior to perform ANOVA (*P* < 0.05). Ratio in parenthesis refers to PTX:DXR molar ratio. Abbreviations: DXR, doxorubicin; PTX, paclitaxel; LI + I, large irregular plus irregular; LR, large regular; N, normal; SR, small regular.

**Figure 3:**
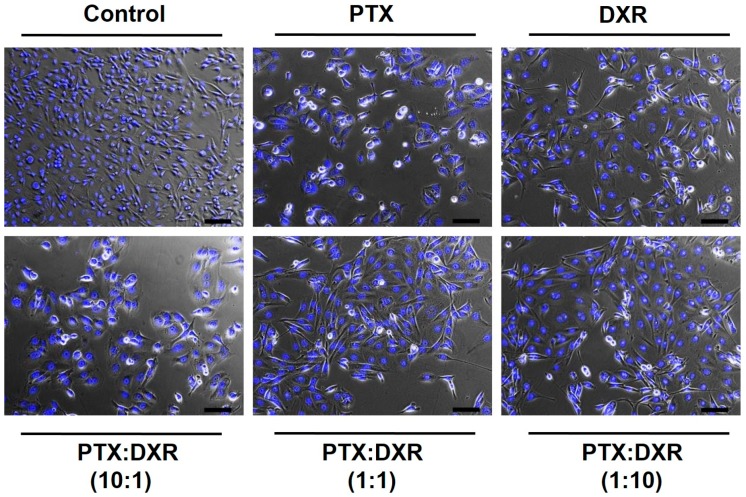
Merge of phase-contrast and fluorescence photomicrographs of MDA-MB-231 cell line after different treatments at concentration of 70 nM for 48 h. In the control group, cell shapes were intact and with higher density. On all treated groups, it was observed enlarged and flattened cells, matching the phenotypic morphology of senescence. Round cells, characteristic of mitotic arrest can be observed on groups treated with PTX and PTX:DXR at 10:1 molar ratio. Nuclei were stained with Hoescht 33342. Images are representative of three independent experiments. Amplification 10×, scale bar = 100 μm. Abbreviations: DXR, doxorubicin; PTX, paclitaxel.

The morphometric analysis of the nuclei size and irregularity showed that all three cells lines responded in a similar manner to the different treatments, which resulted predominantly in an increase of the percentage of LR nuclei, characteristic of senescence, as shown in Figure 2. Cancer cell senescence is a state of cellular arrest that frequently occurs in response to therapy. It is often called therapy-induced senescence (TIS) or accelerated cellular senescence, to differentiate from the aging process of normal cells known as replicative senescence [32,33]. The percentage of LR nuclei for the MDA-MB-231 cell line exposed to the different treatments ranged from 55 to 83%, while that for MCF-7cell line ranged from 37 to 68% and that for SKBR-3 cell line ranged from 51 to 66%. On the phase contrast photomicrographs of MDA-MB-231 cells, shown in Figure 3, we observe enlarged and flattened cells in groups exposed to the different treatments, compared to the control. These characteristics correspond with the typical phenotypic morphology of senescence [34,35].

The different treatments also resulted in nuclear irregularities, characteristic of mitotic catastrophe or another nuclear damaging event, in a similar extension for the three cell lines. The percentage of LI + I nuclei for the MDA-MB-231 cell line exposed to the different treatments ranged from 5 to 27%, while that for MCF-7 and SKBR-3 cell lines ranged from 6 to 25% and 8 to 24%, respectively. For MDA-MB-231 and MCF-7 cell lines, the higher percentage of LI + I nuclei was observed after treatment with PTX while for SKBR-3 cell line when the cells were exposed to PTX:DXR at 10:1 molar ratio. A higher proportion of nuclear irregularities for treatments containing higher concentrations of microtubule-hyperpolymerizing agents, such as PTX, is expected since these drugs result in damage further leading to mitotic catastrophe [36,37,38]. The presence of cells with a round shape, characteristic of mitotic arrest, is evident for MDA-MB-231 cells exposed to PTX or PTX:DXR at 10:1 molar ratio, as shown in Figure 3. When the drugs are combined in equimolar concentrations it is possible to verify the dominance of the effect produced by DXR treatment, preventing the effects of the PTX treatment in morphology. Similar results have been published by Blagosklonny and coworkers for colon cancer cells exposed sequentially to DXR treatment followed by PTX treatment [39]. The presence of SR nuclei, characteristic of apoptosis was just observed for MDA-MB-231 cell line when treated with PTX (1%) and PTX:DXR at molar ratio of 1:1 (2%).

### 3.4. Senescence-Associated-β-galactosidase (SA-β-gal) Assay

Senescent cells express SA-β-gal activity that is detectable at pH 6.0, distinct from the acidic β-galactosidase activity, present in all cells and detectable at pH 4.0. SA-βgal can be detected using chromogenic substrate X-gal, which yields an insoluble blue compound when cleaved by β-galactosidase [28].

Usually the technique consists in SA-β-gal staining followed by the visual identification of the positive cells. This process is considered to be much more subjective and time consuming than using the NMA tool, previously demonstrated to be useful in predicting the senescent status of a cell population [31]. Therefore, in the present study the SA-βgal assay was performed only qualitatively aiming to confirm the NMA findings. 

**Figure 4:**
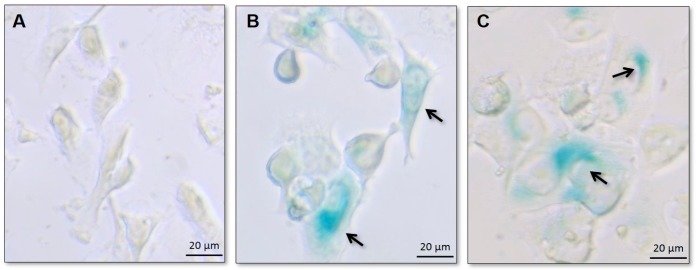
Bright field photomicrographs of MDA-MB-231 cell line of the control group (**A**) or after treatments with PTX (**B**) and DXR (**C**) at a concentration of 70 nM for 48 h. On treated groups, cells are larger and were positively stained for SA-β-gal as indicated by arrows, confirming the occurrence of senescence. Amplification 40×.

For all three cell lines, cells of the control group were not stained by X-gal, as expected. Blue-stained cells were present in cell populations exposed to all the different treatments (PTX, DXR, and the mixtures of free PTX:DXR at 10:1; 1:1 or 1:10 molar ratio), confirming the occurrence of senescence. Figure 4 illustrates the results obtained for control, PTX and DXR treated MDA-MB-231 cell line. Similar findings were observed for all the other treatments and cell lines (data not shown). 

### 3.5. Clonogenic Assay

**Figure 5:**
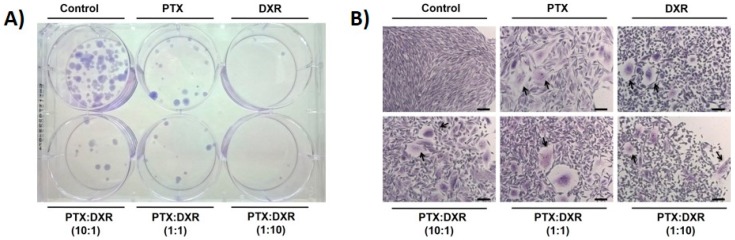
Colonies formed 21 days after re-plating MDA-MB-231 cells previously exposed to 70 nM of different treatments for 48 h. Photograph of plate containing colonies (**A**) and photomicrographs of crystal violet stained colonies, amplification 10×, scale bar = 100 μm. Arrows indicate cells reassuming the senescence morphology (**B**). Abbreviations: DXR, doxorubicin; PTX, paclitaxel. Ratio in parenthesis refers to PTX:DXR molar ratio.

The clonogenic assay essentially tests every cell of the population for its ability to undergo ‘‘unlimited’’ division. It consists in a method to evaluate the long-term effectiveness of cytotoxic agents [28]. The ability of a single cell to grow into a large colony that can be visualized by the naked eye, as presented for MDA-MB-231 cell line on Figure 5A, is proof that it has retained its capacity to reproduce [40].

Figure 5B presents photomicrographs of crystal violet stained colonies of MDA-MB-231 cell line growing after the exposure to the different treatments at 70 nM. Cells of the colonies of the control group presented the characteristic morphology of MDA-MB-231 cell line, with spindle-shaped cells. Cells of colonies of previously treated groups consist of pleomorphic cell populations. Colonies from cells previously exposed to free PTX or the mixture of free PTX:DXR at 10:1 molar ratio were enlarged morphologically when compared to colonies from cells previously exposed to free DXR or the mixtures of free PTX:DXR at 1:1 and 1:10 molar ratios. Similarly to the findings shown after exposition to the treatments for 48 h (Figure 3), when the drugs are combined at equimolar concentrations it is possible to verify the predominance of the effect of the DXR treatment, that also prevented the effects of PTX on the morphology of cells that recovered from treatment. For all treated groups, cells reassuming the senescence morphology could be verified in the colonies and are indicated by arrows in Figure 5B.

**Figure 6:**
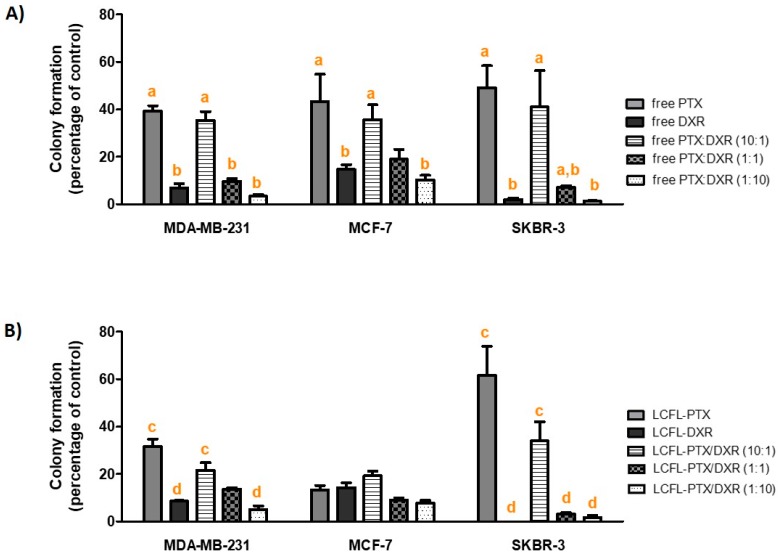
Percentage of colony formation in relation to the control for the different cell lines evaluated when exposed to different ratios of free (**A**) or liposome co-encapsulated PTX and DXR (**B**) at total concentration of 70 nM for 48 h. Data for MDA-MB-231 and MCF-7 cell lines were transformed as *y* = log(value) and for SKBR-3 cell line as *y* = log(value + 1) prior to ANOVA. ^a^ differs statistically from free DXR treatment; ^b^ differs statistically from free PTX treatment; ^c^ differs statistically from LCFL-DXR treatment; ^d^ differs statistically from LCFL-PTX treatment. Ratio in parenthesis refers to PTX:DXR molar ratio. Abbreviations: DXR, doxorubicin; PTX, paclitaxel; LCFL, long-circulating and fusogenic liposomes; LFCP-PTX/DXR, long- circulating and fusogenic liposomes co-encapsulating paclitaxel and doxorubicin.

As shown in Figure 6, considering the treatments on the free form, all cell lines presented lower percentages of colony formation when exposed to DXR compared to PTX (*P* < 0.05). This lower percentage of colony formation was also observed for MDA-MB-231 and SKBR-3 cell lines exposed to LCFL-DXR compared to LCFL-PTX (*P* < 0.05), while no difference between these treatments was observed for MCF-7 cell line (*P* > 0.05).

In general, the encapsulation of the different treatments did not affect significantly the percentage of colony formation for the different cell lines. Exceptions consisted in the encapsulation of PTX for the MCF-7 cell line, and the encapsulation of PTX:DXR at 1:1 molar for the SKBR-3 cell line, both of which led to a significant reduction of colony formation on these cell lines (*P* < 0.05).

#### Comparison of the Long-Term Cytotoxicities of the Different Combinations of PTX:DXR

Despite the different response profiles observed for the short-term cytotoxicity analyses, all three cell lines behaved similarly in the clonogenic assay. This assay revealed that despite the higher cytotoxicity observed for PTX treatment compared to DXR treatment (either in free form or encapsulated in liposomes) when evaluated for its short-term effect, the cells that remained after 48 hours of treatment with this drug retained a better ability to proliferate compared to the cells treated with DXR. In these experiments, higher concentrations of DXR in the combinations led to the lower percentages of colony formation for the three different cell lines. Considering combinations of PTX:DXR on the free form at 1:1 and 1:10 molar ratios, we observed a reduction level of the percentage of colony formation equal to 3.7-fold and 10.7-fold, respectively, compared to the 10:1 molar ratio against the MDA-MB-231 cell line. For the MCF-7 cell line, the 1:1 and 1:10 molar ratios of PTX:DXR reduced 1.9-fold and 3.4-fold, respectively, the percentage of colony formation compared to the 10:1 molar ratio. In the case of the SKBR-3 cell line, this reduction in percentage of colony formation was of 5.7-fold and 32.8-fold for the 1:1 and 1:10 molar ratios respectively, compared to the 10:1 molar ratio. When comparing the combinations of the free drugs at 1:1 and 1:10 molar ratios, the 1:10 ratio allowed for a significant reduction of colony formation compared to the 1:1 ratio for the MDA-MB-231 and SKBR-3 cell lines (*P* < 0.05), while no difference was observed between these ratios in the MCF-7 cell line (*P* > 0.05). Considering the co-encapsulated combinations of PTX:DXR, the 1:1 and 1:10 molar ratios reduced 1.6-fold and 4.2-fold, respectively, the percentage of colony formation compared to the 10:1 molar ratio against the MDA-MB-231 cell line. For the MCF-7 cell line, the 1:1 and 1:10 molar ratios reduced on 2.1-fold and 2.5-fold, respectively, the percentage of colony formation compared to the 10:1 molar ratio. This reduction in percentage of colony formation was of 11.2-fold and 20.2-fold for the 1:1 and 1:10 molar ratios, respectively, compared to the 10:1 molar ratio in the case of the SKBR-3 cell line. When comparing the 1:1 and 1:10 ratio combinations of the co-encapsulated drugs, the 1:10 ratio allowed for a significant reduction of colony formation compared to the 1:1 ratio for the MDA-MB-231 cell line (*P* < 0.05), while no difference was observed for these ratios on the MCF-7 and SKBR-3 cell lines (*P* > 0.05). 

### 3.6. Migration Assay

**Figure 7:**
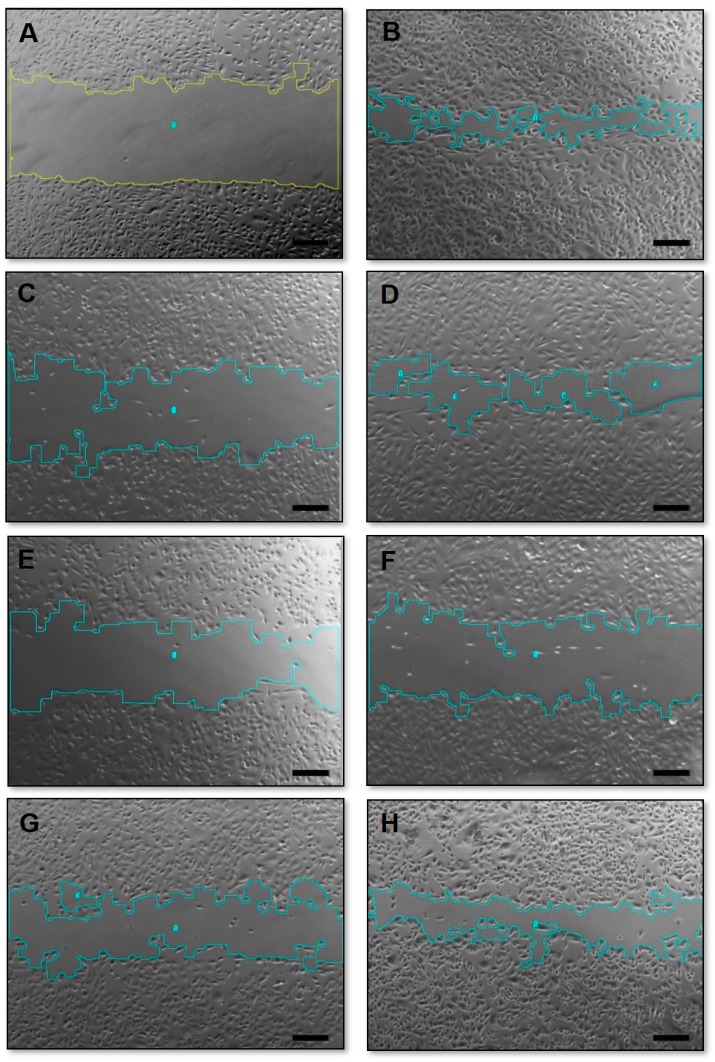
Phase-contrast photomicrographs of the “zero wound” (**A**) and wounds of MDA-MB-231 cell line on control group (**B**); exposed for 24 h to 70 nM of PTX (**C**); DXR (**D**); PTX:DXR combinations at 10:1 (**E**); 1:1 (**F**); or 1:10 (**G**) molar ratios and to 6.4 nM of PTX (**H**). Amplification 5×, scale bar = 200 μm.

The wound healing assay allows the observation of two-dimensional (2D) cell migration in confluent, monolayer cell cultures [41]. When performing migration assays, it is important to guarantee that the wound closures are due to the cell migration and not proliferation. Therefore, experiments in this work were performed in lower FBS concentration (1%), a procedure known as cell starvation, which suppresses cellular proliferation [42]. In addition, the wound closures were evaluated 24 h after exposure to the different treatments once longer study periods do not allow distinguishing between cell proliferation and changes in cell survival from cell motility [43]. Representative phase-contrast photomicrographs of the scratches after 24 h exposure to the treatments are presented in Figure 7.

As summarized in Table 3, the treatment with free or liposome encapsulated DXR did not affect the cell migration for MDA-MB-231 or MCF-7 cell lines, which did not differ statistically from migration observed for the control group (*P* > 0.05). All treatments containing PTX reduced significantly the percentage of cell migration compared to the control group for both cell lines (*P* < 0.05), which was expected as microtubule-affecting drugs are known to present significant anti-migration properties [44].

Considering combinations of free PTX and DXR in different molar ratios, difference was observed in themigration percentage only between PTX:DXR treatments at molar ratio equal to 1:1 and 1:10 using MDA-MB-231 cell line (*P* < 0.05). For MCF-7 cell line, all molar ratios of free drug combinations reduced migration percentage to the same extent (*P* > 0.05). When co-encapsulated in liposomes, all different molar ratios of PTX and DXR reduced migration percentage to the same extent for both cell lines (*P* > 0.05).

It is interesting to note that even the addition of a small proportion of PTX, as in the 1:10 PTX:DXR molar ratio combination, allowed for a significant reduction in the migration percentage of both cell lines when compared to the treatment with DXR alone, either in free form or co-encapsulated in liposomes. In the 1:10 PTX:DXR molar ratio combination the concentrations of PTX and DXR are 6.36 and 63.63 nM, respectively. In order to verify if a PTX concentration of 6.4 nM was sufficient to inhibit cell migration to the same extent as observed for the 1:10 combinations, we tested this dose either free or encapsulated in liposomes against the two cell lines. For the MDA-MB-231 cell line, cell migration percentages after exposure to free PTX or LCFL-PTX at a 6.4 nM concentration were 98.1 ± 0.1% and 76.4 ± 6.9%, respectively, differing significantly from those observed for the combined 1:10 treatments (*P* < 0.05). For the MCF-7 cell line, cell migration percentages after exposure to free PTX or LCFL-PTXPTX at 6.4 nM concentration were 74.8 ± 3.1% and 72.6 ± 1.5%, respectively, and differed significantly from those observed for the combined 1:10 treatments (*P* < 0.05). Thus, these findings show that the cellular migration inhibition observed for the 1:10 molar ratio combination is not due to an effect of the PTX alone, suggesting a potentiation effect when the treatment was performed with PTX and DXR together.

## 4. Discussion

We described herein the results of short- and long-term experiments aiming to evaluate the effects of PTX, DXR, or its combinations either free or co-encapsulated in liposomes in different molar ratios against three human breast cancer cell lines. 

Different studies from the 1990s reported results of in vitro studies of PTX:DXR combinations suggesting antagonism between the agents. However, the available data are controversial since cytotoxic interaction appeared to be schedule and cell line dependent. These studies were mainly short-term experiments and, at the time, did not take into account the possible molar ratio dependency effects of the combination [45,46,47,48]. On the contrary of these in vitro studies, the clinical experience accumulated in the following years shows that adding a taxane to an anthracycline based regimen is beneficial to the patients with early breast cancer [3,4]. Nowadays, combinations of PTX and DXR are extensively used in the clinics following protocols that indicate the administration of both drugs in their maximum tolerated dose (MTD) [3]. As these protocols do not take into account the possible differences on efficacy due to the ratio of the drugs, it is clear that we might be failing to exploit the full therapeutic potential of this combination as well as leading to unnecessary toxicity. In fact, recent studies evaluating different molar ratios of PTX:DXR combinations reported synergism for combinations on which DXR is present in higher proportions compared to PTX [17,49,50,51]. Moreover, another important point to consider is the warranty of delivery of the drug ratio that is active to the region of interest. The co-encapsulation of synergistic PTX:DXR molar ratios in nanosystems enables its delivery to the tumor site, which cannot be done upon the administration of the free drugs. This idea has been validated on in vivo studies that demonstrated the superiority of synergistic ratios of PTX:DXR co-encapsulated in drug delivery systems compared to the mixture of the free drugs at the same ratio, or to a co-encapsulated antagonistic ratio of the drugs [17,51].

Taken together, our results are in accordance with these data and allow us to suggest that there is no benefit in enhancing the PTX concentration above that of DXR in the combination for any of the three cell lines tested. In short-term cytotoxicity studies, synergism was verified for MDA-MB-231 and MCF-7 cell lines only when PTX and DXR were combined at 1:10 molar ratio, while none of the combinations was synergistic for the SKBR-3 cell line. The clonogenic assay revealed that in a long-term evaluation, DXR-based treatments were much more efficient at inhibiting colony formation compared to PTX-based treatments. Further studies are necessary to elucidate the mechanisms behind the better recovery ability of the cells treated with higher amounts of PTX in the present work. The evaluation of the crystal violet stained colonies under the microscope and the NMA allow us to propose two lines of investigation. First, treatments with PTX or PTX:DXR at 10:1 molar ratio had at least a tendency to lead to higher percentages of nuclear irregularities compared to the treatments with DXR alone or PTX:DXR at molar ratios of 1:1 and 1:10, as indicated by NMA. These irregularities indicate mitotic arrest as expected for treatment with PTX. One possibility is that some of these cells are going through a process known as “mitotic slippage”, which allows the cells to escape from apoptosis. A small fraction of these slipped cells is believed to continue dividing, thus resisting to the treatment and that could explain the higher percentage of colony formation observed when cells were exposed to PTX-based treatments [52,53,54].

The other possibility is that cells exposed to PTX-based treatments might be escaping more efficiently from senescence compared to cells exposed to DXR-based treatments, thus leading to a higher percentage of colony formation. Cells reassuming the senescence morphology could be verified in the colonies of all treated groups. It has been stated by Roberson and co-workers (2005) that such cells are derived from TIS cells that circumvented senescence [55]. TIS was initially believed to be an irreversible cell-cycle arrest; however, a growing line of evidence identifies it as a reversible state. A recent review published by Chakradeo and coworkers lists the over-expression of Cdc2/cdk1 and survivin as one of the possible pathways for TIS escape [56]. The up-regulation of Cdc2/cdk1 in cells after exposure to PTX treatment has already been reported for different cell lines. This up-regulation leads to the phosphorylation and accumulation of survivin, believed to be the downstream effector of TIS escape [57,58]. Different studies have reported that the treatment of MCF-7 cells with PTX rapidly up-regulated survivin expression within 4–6 h, in a process that was independent from G2/M arrest [59,60]. Bridget and coworkers have shown that the treatment of MDA-MB-231 cells with PTX caused them to generate exosomes highly enriched with survivin. The treatment of this cell line with DXR did not lead to the same fate [61]. Indeed, exposure of MDA-MB-231 and MCF-7 cell lines to DXR treatment has been previously reported to lead to a reduction on survivin levels [62].

It has been suggested, but not confirmed, that the source of recurrent disease in the clinics might be an initially senescent tumor cell population [56]. The occurrence of TIS in breast tumors of patients who received cyclophosphamide, doxorubicin, and fluorouracil (CAF) regimen was demonstrated by Poele and coworkers. For patients who received adjuvant chemotherapy, 15 of 36 tumors (41%) were positively stained for senescence-associated β-galactosidase, while only 2 of 20 tumors (10%) from patients who underwent surgery without chemotherapy were positive for this marker [63]. Treatments that lead to better elimination of senescent cells, as DXR-based treatments in this work, are highly desirable. 

Another important hallmark evaluated was cell migration. Although the metastasis event involves multiple processes, it is strictly connected to the initial cell migration and tissue invasion from the primary tumor site [43]. The migration assay showed that the DXR treatment alone was not able to inhibit cell migration but the addition of small amounts of PTX to the DXR treatment allowed a significant inhibition of this migration. Considering that metastasis represents the major problem in the treatment of cancer, being indicative of poor prognosis and having dramatic effects on the survival of patients, the effects of the association of PTX to DXR treatment on cell migration here presented are important, emphasizing the benefits of the combination [43].

In our previous study with Balb/c mice bearing 4T1 murine mammary carcinoma we report that the addition of small amounts of PTX to DXR (1:10 molar ratio) is enough to significantly enhance treatment activity (compared to DXR alone) and that the LCFL-PTX/DXR improves the cardiac toxicity profile compared to treatment with the free combination of these drugs [21]. These previous findings together with the present evaluation in human breast cancer cell lines allow us to suggest that it might be possible to reduce the PTX dose used in the clinics, retaining the efficacy of the combination, as long as the expected molar ratio between the drugs reaches the tumor site. In addition, it is known that the PTX administration alters DXR pharmacokinetics, as a result of an interference with its elimination, enhancing the plasma concentration of DXR and its metabolite, doxorubicinol, also known to be highly cardiotoxic [64]. This pharmacokinetic interference leads to an enhancement of the combination’s cardiotoxicity, demanding the administration of PTX and DXR to be separated, and limiting the DXR cumulative dose possible to be administered to the patients [65]. Thus, the decrease of the PTX dose in the regimens containing the combination DXR and PTX might reduce this interference and have a good impact on toxicity [64]. As previously mentioned, it is impossible to deliver a pre-determined ratio of drugs to the tumor site upon the administration of free drugs due to the dissimilar pharmacokinetics of most agents. For this reason, we also obtained and evaluated the effects of LCFL-PTX/DXR co-encapsulating different PTX:DXR molar ratios. In general, the encapsulation of different PTX:DXR molar ratios in LCFL did not impact the biological activity of the drugs. Thus, LCFL-PTX/DXR are a promising strategy to allow the delivery of optimal PTX:DXR molar ratios to the tumor site, which according to this and the previously cited studies include a reduced dose of PTX. The reduction of the PTX concentration in the combination itself might lead to a reduction in toxicity. Besides, co-encapsulating the drugs avoids the pharmacokinetics interactions between PTX and DXR, enabling the concomitant administration of PTX and DXR, which is suggested to have better efficacy than the administration of these drugs with an interval [66,67].

## 5. Conclusions

Chemotherapy regimens containing PTX and DXR are pivotal in the management of BC [3,4]. Nowadays, the protocols are still developed based on the same principles that Frei and coworkers proposed in the 1960s, including administration of the drugs on their MTD [9]. The results presented herein are in accordance with previous studies that show that the ratio of PTX and DXR in the combination plays an important role in the biological activity. These results allow us to suggest that there is no benefit in enhancing the PTX concentration above that of DXR in the combinations. Since the only way to deliver a pre-determined ratio of drugs to the tumor site is through the co-encapsulation, we developed LCFL-PTX/DXR which retained the biological properties of the mixture of free drugs. This formulation can be a promising strategy for the management of BC as it is designed to deliver an optimal ratio of the PTX:DXR combination to the tumor site.

## Figures and Tables

**Table 1:** Physicochemical characteristics of different liposomal formulations.

Liposomal Formulation	[PTX] (mg/mL)	[DXR] (mg/mL)	PTX/DXR Molar Ratio	Mean Diameter (nm)	PDI	Zeta Potential (mV)
LCFP-Blank	-	-	-	228.5 ± 10.14	0.33 ± 0.06	−5.25 ± 0.67
LCFP-PTX	0.12 ± 0.01	-	-	247.0 ± 4.3	0.31 ± 0.04	−5.07 ± 0.65
LCFP-DXR	-	1.72 ± 0.39	-	226.4± 26.9	0.27± 0.03	−4.42 ± 1.38
LCFP-PTX/DXR (10:1)	0.13 ± 0.02	0.01 ± 0.00	9.82 ±0.24	249.8 ± 12.3	0.29 ± 0.01	−6.86 ± 1.67
LCFP-PTX/DXR (1:1)	0.12 ± 0.02	0.08 ± 0.01	0.98 ± 0.07	236.3 ± 5.1	0.29 ± 0.02	−6.40 ± 1.78
LCFP-PTX/DXR (1:10)	0.14 ± 0.03	0.81 ± 0.14	0.11 ± 0.05	237.1 ± 48.3	0.29 ± 0.04	−4.40 ± 1.31

Ratio in parenthesis refers to PTX:DXR molar ratio. Abbreviations: DXR, doxorubicin; PTX, paclitaxel; LCFL, long-circulating and fusogenic liposomes; LCFL-PTX/DXR, long-circulating and fusogenic liposomes co-encapsulating paclitaxel and doxorubicin; PI, polydispersity index.

**Table 2:** Values of *IC*_50_ and *CI* obtained for the different cell lines evaluated when exposed to different ratios of free or liposome co-encapsulated PTX and DXR for 48 h.

Treatment	MDA-MB-231		MCF-7		SKBR-3	
	*IC*_50_ (μM)	*CI*	*IC*_50_ (μM)	*CI*	*IC*_50_ (nM)	*CI*
Free PTX	0.67 ± 0.19 ^a^	-	0.31 ± 0.04 ^a^	-	0.99 ± 0.22 ^a^	-
Free DXR	3.01 ± 0.37 ^b^	-	1.31 ± 0.11 ^b^	-	144.19 ± 17.42 ^b^	-
Free PTX:DXR (10:1)	9.73 ± 3.01 ^a,b^	13.32 ± 4.12	>5.00	N.D.	1.47 ± 0.69 ^a^	1.36 ± 0.64
Free PTX:DXR (1:1)	2.09 ± 0.36 ^b^	1.91 ± 0.32	0.98 ± 0.23 ^b^	2.05 ± 0.49	4.40 ± 0.48 ^a,b^	2.27 ± 0.25
Free PTX:DXR (1:10)	1.81 ± 0.54 ^b^	0.82 ± 0.25	0.88 ± 0.03 ^b^	0.87 ± 0.03	57.41 ± 8.14 ^a,b^	5.69 ± 0.81
LCFL-PTX	1.13 ± 0.26	-	0.16 ± 0.05 ^c^	-	2.67 ± 1.03 ^c^	-
LCFL-DXR	1.84 ± 0.20	-	1.85 ± 0.40 ^d^	-	196.08 ± 28.08 ^d^	-
LCFL-PTX/DXR (10:1)	7.99 ± 2.27 ^c,d^	7.25 ± 2.07	>5.00	N.D.	20.29 ± 5.06 ^c,d^	7.41 ± 1.89
LCFL-PTX/DXR (1:1)	6.05 ± 0.98 ^c,d^	4.50 ± 0.72	1.23 ± 0.45 ^c,d^	4.55 ± 1.63	27.22 ± 6.38 ^c,d^	5.52 ± 1.29
LCFL-PTX/DXR (1:10)	3.79 ± 0.77 ^c,d^	2.20 ± 0.45	0.77 ± 0.22 ^c,d^	0.84 ± 0.02	120.22 ± 31.68 ^d^	4.93 ± 1.30

Data were transformed as *y* = log(value) prior to ANOVA. ^a^ differs statistically from free DXR treatment; ^b^ differs statistically from free PTX treatment; ^c^ differs statistically from LCFL-DXR treatment; ^d^ differs statistically from LCFL-PTX treatment; underlined values = differs statistically from equivalent non-encapsulated treatment. Ratio in parenthesis refers to PTX:DXR molar ratio. Abbreviations: CI, combination index; DXR, doxorubicin; PTX, paclitaxel; IC_50_, inhibitory concentration of 50%; LCFL, long-circulating and fusogenic liposomes; LCFP-PTX/DXR, long-circulating and fusogenic liposomes co-encapsulating paclitaxel and doxorubicin; PI, polydispersity index.

**Table 3:** Percentage of cellular migration in relation to the control for the different cell lines evaluated when exposed to different ratios of free or liposome co-encapsulated PTX and DXR at total concentration of 70 nM for 24 h.

Treatment	MDA-MB-231	MCF-7
Free PTX	30.4 ± 6.7 ^a^	64.3 ± 2.6 ^a^
Free DXR	101.0 ± 9.6 ^b^	110.3 ± 6.8 ^b^
Free PTX:DXR (10:1)	42.5 ± 11.1 ^a^	56.6 ± 3.6 ^a^
Free PTX:DXR (1:1)	40.4 ± 9.2 ^a^	63.4 ± 2.8 ^a^
Free PTX:DXR (1:10)	62.8 ± 5.2 ^a,b^	59.9 ± 1.7 ^a^
LCFL-PTX	38.6 ± 11.9 ^c^	47.9 ± 0.8 ^c^
LCFL-DXR	85.2 ± 13.0 ^d^	112.3 ± 3.0 ^d^
LCFL-PTX/DXR (10:1)	21.6 ± 4.6 ^c^	52.4 ± 1.4 ^c^
LCFL-PTX/DXR (1:1)	37.2 ± 10.0 ^c^	59.1 ± 7.4 ^c^
LCFL-PTX/DXR (1:10)	40.5 ± 9.4 ^c^	52.0 ± 6.4 ^c^
Free PTX 6.4 nM	98.1 ± 0.1 ^e^	74.8± 3.1 ^e^
LCFL-PTX 6.4 nM	76.4 ± 6.9 ^f^	72.6 ± 1.5 ^f^

Data were transformed as *y* = square root(value) prior to ANOVA. ^a^ differs statistically from free DXR treatment; ^b^ differs statistically from free PTX treatment; ^c^ differs statistically from LCFL-DXR treatment; ^d^ differs statistically from LCFL-PTX treatment; ^e^ differs statistically from free PTX:DXR (1:10) treatment; ^f^ differs statistically from LCLF-PTX/DXR (1:10) treatment; underlined values = differs statistically from equivalent non-encapsulated treatment. Ratio in parenthesis refers to PTX:DXR molar ratio. Abbreviations: DXR, doxorubicin; PTX, paclitaxel; LCFL, long-circulating and fusogenic liposomes; LFCP-PTX/DXR, long-circulating and fusogenic liposomes co-encapsulating paclitaxel and doxorubicin; PI, polydispersity index.

## Supplementary Materials

The following are available online at https://www.mdpi.com/1999-4923/11/4/178/s1, Figure S1: Intensity particle size distribution for LCFL-Blank (**A**), LCFL-PTX (**B**), LCFL-DXR (**C**), LCFL-PTX/DXR 10:1 (**D**), LCFL-PTX/DXR 1:1 (**E**), LCFL-PTX/DXR 1:10 (**F**), Figure S2: Cell viability curves for MDA-MB-231 (panel **A**); MCF-7 (panel **B**) and SKBR-3 (panel **C**) cell lines when exposed to different ratios of free or liposome co-encapsulated PTX and DXR for 48 h.
