# Supplementary material for: Short and Long-Term Effects of the Exposure of Breast Cancer Cell Lines to Different Ratios of Free or Co-Encapsulated Liposomal Paclitaxel and Doxorubicin
Source: Pharmaceutics. 2019 Apr 11;11(4):178. doi: 10.3390/pharmaceutics11040178 (PMC6523953; doi:10.3390/pharmaceutics11040178)
Supplement: Supplementary file 1 [file pharmaceutics-11-00178-s001.pdf]

Supplementary Materials: Short and Long-Term Effects of the Exposure of Breast Cancer Cell Lines to Different Ratios of Free or Co-Encapsulated Liposomal Paclitaxel and Doxorubicin

Marina Santiago Franco, Marjorie Coimbra Roque and Mônica Cristina Oliveira

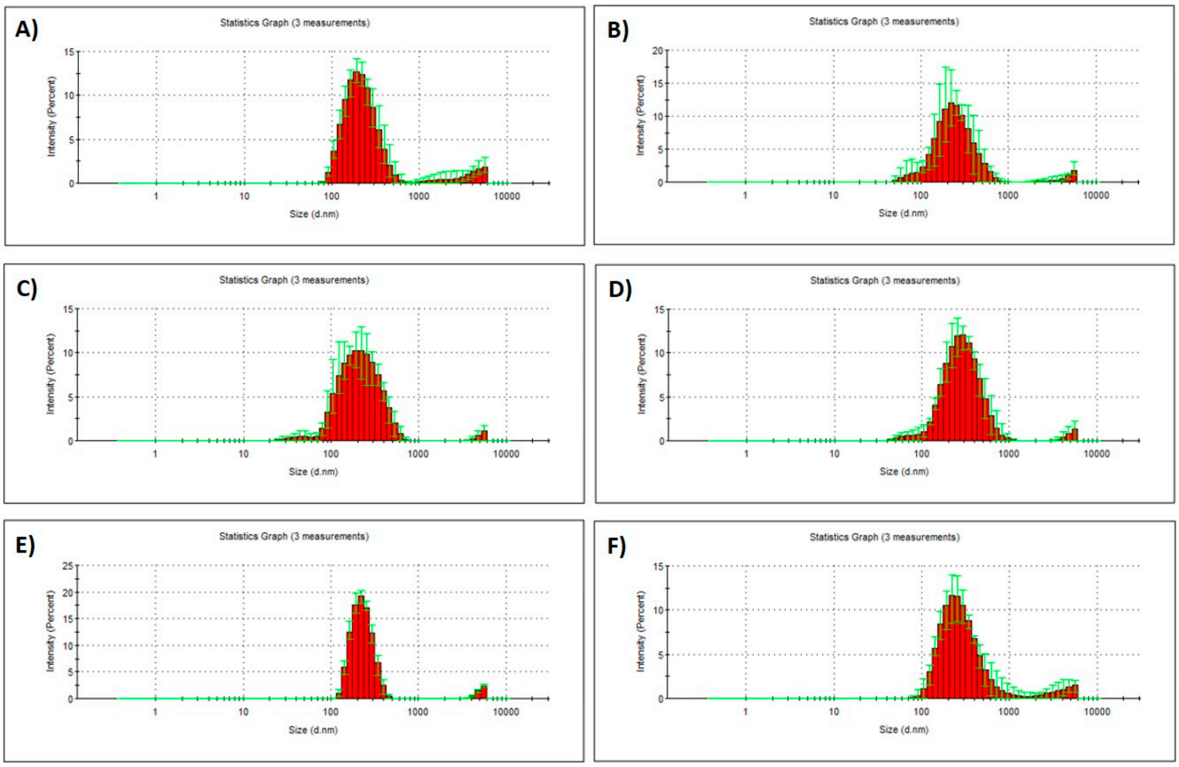

Figure S1. Intensity particle size distribution for LCFL-Blank (**A**), LCFL-PTX (**B**), LCFL-DXR (**C**), LCFL-PTX/DXR 10:1 (**D**), LCFL-PTX/DXR 1:1 (**E**), LCFL-PTX/DXR 1:10 (**F**). Ratio refers to PTX:DXR molar ratio. Abbreviations: DXR, doxorubicin; PTX, paclitaxel; LCFL, long-circulating and fusogenic liposomes; LCFL-PTX/DXR, long-circulating and fusogenic liposomes co-encapsulating paclitaxel and doxorubicin.

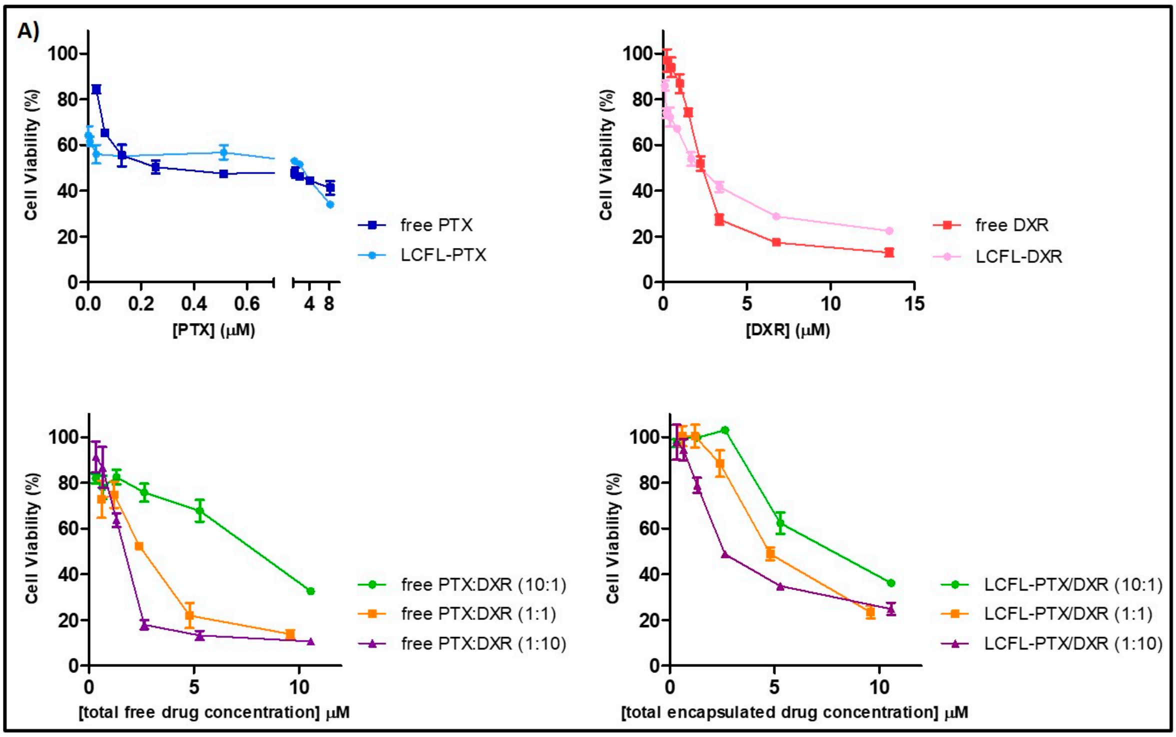
A)
Cell Viability (%)
[PTX] (μM)
free PTX
LCFL-PTX
Cell Viability (%)
[DXR] (μM)
free DXR
LCFL-DXR
Cell Viability (%)
[total free drug concentration] μM
free PTX:DXR (10:1)
free PTX:DXR (1:1)
free PTX:DXR (1:10)
Cell Viability (%)
[total encapsulated drug concentration] μM
LCFL-PTX/DXR (10:1)
LCFL-PTX/DXR (1:1)
LCFL-PTX/DXR (1:10)

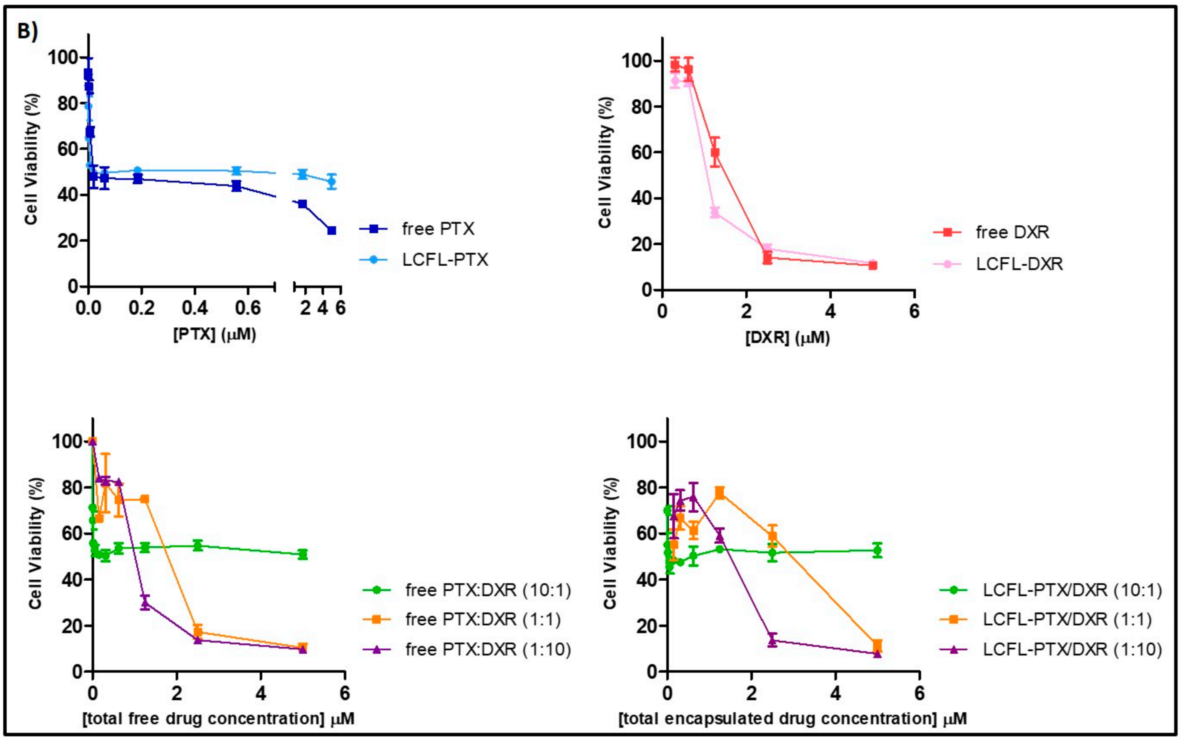
B)
Cell Viability (%)
[PTX] (μM)
free PTX
LCFL-PTX
Cell Viability (%)
[DXR] (μM)
free DXR
LCFL-DXR
Cell Viability (%)
[total free drug concentration] μM
free PTX:DXR (10:1)
free PTX:DXR (1:1)
free PTX:DXR (1:10)
Cell Viability (%)
[total encapsulated drug concentration] μM
LCFL-PTX/DXR (10:1)
LCFL-PTX/DXR (1:1)
LCFL-PTX/DXR (1:10)

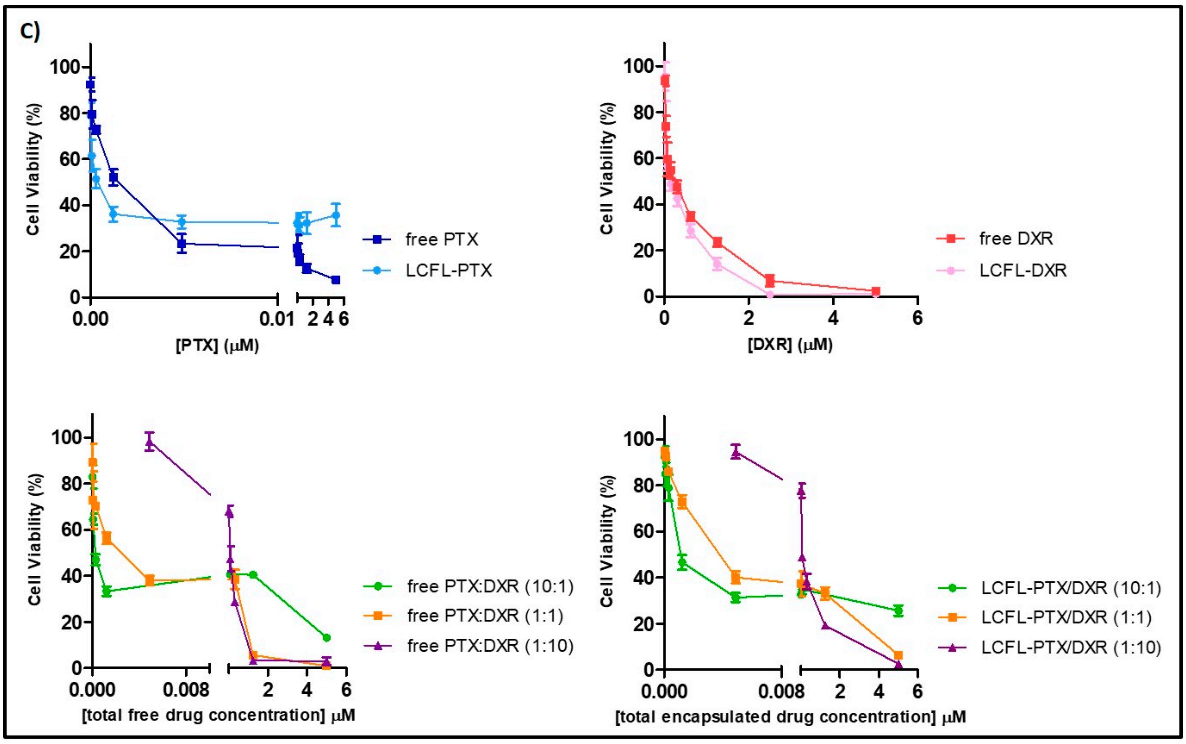

Figure S2. Cell viability curves for MDA-MB-231 (panel **A**); MCF-7 (panel **B**) and SKBR-3 (panel **C**) cell lines when exposed to different ratios of free or liposome co-encapsulated PTX and DXR for 48 h.
